# Double-blind randomised trial of saline solution for gargling and nasal rinsing in SARS-CoV-2 infection

**DOI:** 10.7189/jogh.14.05044

**Published:** 2024-12-30

**Authors:** Sebastian R Espinoza, Lexton Trauffler, Amir Shamshirsaz, Alireza Shamshirsaz, Andres Espinoza, Jimmy Espinoza, Alice O'Brien

**Affiliations:** 1 Trinity University, San Antonio, Texas, USA; 2 UTHealth|McGovern Medical School, Houston, Texas, USA; 3 Department of Obstetrics and Gynecology, Baylor College of Medicine, Houston, Texas, USA; 4 Department of Obstetrics and Gynecology, Harvard Medical School, Boston, Massachusetts, USA; 5 Department of Surgery, Baylor College of Medicine, Houston, Texas, USA; 6 Department of Obstetrics and Gynecology, Division of Fetal Intervention, McGovern Medical School, Houston, Texas, USA; 7 Department of Anesthesiology, Critical Care and Pain Medicine, UTHealth, Houston, Texas, USA

## Abstract

**Background:**

Previous studies have shown that hypertonic saline nasal irrigation and gargling reduced the duration of symptoms in upper respiratory infections caused by coronavirus. This study aims to investigate the effects of two saline regimens on symptoms associated with severe acute respiratory syndrome coronavirus-2 (SARS-CoV-2).

**Methods:**

Between 2020 and 2022, individuals aged 18–65 years who tested positive for SARS-CoV-2 infection via polymerase chain reaction (PCR) were randomly assigned to either low- or high-saline regimens for 14 days. The low-saline solutions contained 2.13 g of salt dissolved in eight ounces of warm water, while the high-saline solution contained six grams of salt dissolved in eight ounces of warm water. Participants gargled and rinsed their nasal passages four times a day for 14 days. Primary outcomes assessed included frequency and duration of SARS-CoV-2 symptoms, while secondary outcomes included hospital or intensive care unit (ICU) admission, need for mechanical ventilatory support, or mortality rates. Exclusion criteria included chronic hypertension or participation in other interventional studies.

**Results:**

Fifty-eight individuals were allocated to the low (n = 27) or high (n = 28) saline regimens; with three lost to follow-up. There were no significant differences in primary or secondary outcomes between these groups. Comparatively, during the study period, 9398 individuals with confirmed SARS-CoV-2 infection by positive PCR test were observed as a reference group. Hospitalisation rates in the low-saline (18.5%) and high-saline (21.4%) regimens were significantly lower than in the reference group (58.8%; *P* < 0.001), while no significant differences were observed in other outcomes among these groups.

**Conclusions:**

Low and high saline regimens for gargling and nasal rinsing show similar effectiveness in reducing the frequency and duration of symptoms related to SARS-CoV-2 infection. Both saline regimens are associated with lower hospitalisation rates compared to individuals not using gargling or nasal rinsing in those infected by SARS-CoV-2.

While severe acute respiratory syndrome coronavirus-2 (SARS-CoV-2) infection is no longer a global health emergency, it still affected around 765 000 000 people, resulting in over 7 000 000 deaths. The coronavirus enters into human cells targeting a specific receptor angiotensin converting enzyme-2, found in certain cells [1]. There is a close genetic similarity between severe acute respiratory syndrome coronavirus (SARS-CoV) and the causative agent of COVID-19, SARS-CoV-2 [2]. Diagnostic testing indicates that simple throat swabs provide sufficient sensitivity in detecting SARS-CoV-2 infection during its early stages [2].

Previous reports suggest that active SARS-CoV-2 virus replication occurs in the upper respiratory tract tissues, unlike SARS-CoV, which is not believed to replicate in this area despite angiotensin converting enzyme-2 expression [2]. The authors noted that peak RNA concentrations from nasopharyngeal swabs were reached earlier in SARS-CoV-2 positive individuals compared to those with SARS-CoV, with viral concentrations being 1000 times higher than individuals with SARS-CoV [2]. Furthermore, the authors tested all nasopharyngeal samples against a panel of typical respiratory viral agents, including Human coronavirus HKU1, Human coronavirus OC43, Human coronavirus NL63, Human coronavirus 229E, influenza virus A and B, rhinovirus, enterovirus, respiratory syncytial virus, human parainfluenza virus 1–4, human metapneumovirus, adenovirus, and human bocavirus. They reported no co-infections in any patient [2].

The presence of high viral loads and successful isolation from early throat swabs suggests potential SARS-CoV-2 replication in upper respiratory tract tissues [2]. Therefore, interventions targeting the upper respiratory tract may reduce viral load, potentially decreasing the frequency and duration of upper respiratory symptoms linked to COVID-19 disease. Recent evidence highlights the efficacy of saline gargling and nasal rinsing in SARS-CoV-2 infection. Two recent randomised trials have shown that normal saline [3] and seawater [4] nasopharyngeal washes are associated with shorter symptom durations and reduced viral loads in COVID-19 infections.

Our study aims to determine the impact of gargling and nasal rinsing using both low- and high-concentration saline solutions on the frequency and duration of symptoms associated with SARS-CoV-2 infection. We hypothesise that gargling and nasal rinsing with low- and high-concentration saline solutions lead to a significant reduction in the frequency and duration of symptoms associated with SARS-CoV-2 infection.

## METHODS

This trial was conducted between August 2020 and July 2022 at Harris Health System in Houston, Texas (Baylor College of Medicine IRB# H-47728). Harris Health System is the public health care safety net provider for the residents of Harris County, Texas. Individuals with positive real-time polymerase chain reaction (RT-PCR) for the SARS-CoV-2 virus in nasopharyngeal swabs and meeting the study’s eligibility criteria were identified in coordination with Harris Health System officials and invited to participate.

### Selection and description of participants

Participants were randomly assigned to treatment groups using simple randomisation procedures (computerised random numbers) by an investigator not clinically involved in the trial. The allocation was blinded to participating individuals, outcome assessors, and data analysts.

Participants were randomly allocated to either:

1) Regimen A: salt water gargle and nasal rinsing using six grams (one teaspoon) of salt in eight ounces of warm water, or

2) Regimen B: salt water gargle and nasal rinsing using 2.13 g (1/3 teaspoon) of salt in eight ounces of warm water.

Gargling and nasal rinsing duration was five minutes each, adjusted based on individual tolerance. Participants in either regimen repeated gargling and nasal rinsing four times a day for 14 days after enrolment.

### Inclusion criteria

Participants had to be between 18–65 years of age. They needed to have a documented SARS-CoV-2 virus infection confirmed through one of the following tests used at the Harris Health System: real-time PCR, cobas® SARS-CoV-2 test, Panther Fusion SARS-CoV-2 Assay (Hologic, Inc.), SARS-CoV-2 reverse transcription real-time PCR, Spert Xpress SARS-CoV-2 Assay (Cepheid, Inc.), or 2019-nCoV rRT-PCR. Participants were required to not be enrolled in other interventional research, although individuals using azithromycin, chloroquine, hydroxychloroquine, or Remdesivir were eligible. Those with any comorbidities were allowed, except for chronic hypertension or renal dysfunction. The study was open to participants of any gender, including pregnant and non-pregnant women. Lastly, participants must have experienced the onset of COVID-19 symptoms within 14 days or less.

### Exclusion criteria

Individuals with chronic hypertension or renal dysfunction, including chronic renal disease, were excluded. Participants requiring hospital admission due to COVID-19-related complications at the start of the study were not eligible. Those with a clinical suspicion of pneumonia or radiological/tomographic evidence of pneumonia were also excluded. Additionally, individuals experiencing hypertension during pregnancy were not eligible for the study.

### Data collection and measurements

The primary objective of this study was to assess the effectiveness of gargling and nasal rinsing with low- and high-concentration saline solutions in reducing the frequency and duration of symptoms associated with SARS-CoV-2 infection. Secondary objectives included comparing the rates of hospital admissions, intensive care unit (ICU) admissions, mechanical ventilator use, and mortality rates between the two study groups.

**Figure 1:**
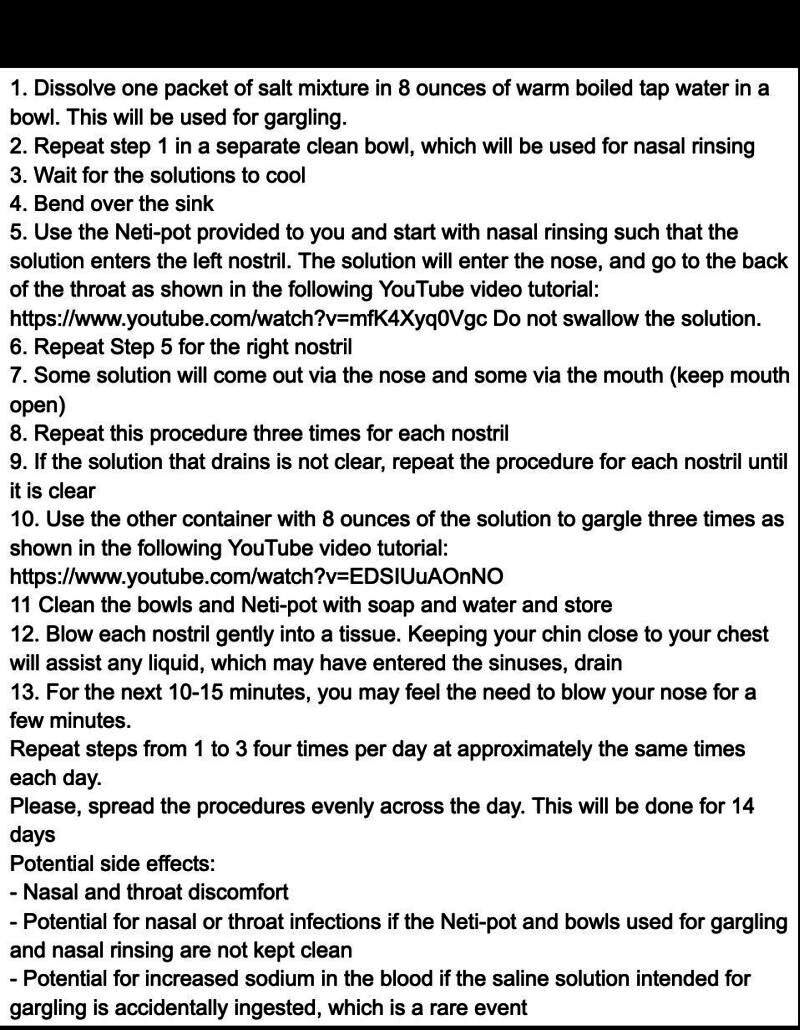
Instructions for preparing the saline solution for saline gargling and nasal rinsing alongside the potential side effects of treatment.

Our coordinating centre shipped boxes containing 112 individually packed doses of salt (four doses for gargling and four for nasal rinsing per day for 14 days) to each patient according to their assigned regimen, along with a Neti-pot. Written instructions were included detailing how to dissolve the salt in warm boiled tap water, and the recommended frequency and duration of salt water gargling and nasal rinsing, following a modified version of a protocol that reported 87% tolerance rate [5] (**Figure 1**).

Participating individuals were provided access to an online RedCap form to record their COVID-19 related symptoms daily. This checklist mirrored those used by health care institutions to monitor for signs or symptoms associated with SARS-CoV-2 virus infection in their workforce. Individuals without online access were contacted by phone by the study coordinator (blinded to which regimen the patient was on) to assess their symptom frequency and duration of the symptoms associated with SARS-CoV-2 infection. The same study coordinator contacted participants every other day (excluding weekends) over a two-week period to ensure proper completion of online forms, record symptoms in those without online access, and document any health care facility admissions. To mitigate selection bias, participants without internet or computer access (for example, those living in transitional halfway homes) were approached by the study coordinator for phone documentation of their disease course. Additional clinical data collected included hospital admissions, ICU admissions, and mechanical ventilator use.

### Statistics

Based on prior research [6], a 44% reduction in symptom duration required enrolment of 50 individuals with a significance level of 0.05 and 90% of power. Anticipating a 10–12% loss to follow-up due to reduced compliance with saline treatment, eight additional participants were enrolled, resulting in 29 individuals allocated to each study arm (total of 58 participants) for intention-to-treat analysis.

Frequency and duration of SARS-CoV-2 virus symptoms were compared between study arms using parametric and non-parametric tests based on data distribution. The Kolmogorov-Smirnov test was used to test the normality of the data. Additionally, comparisons were made between study arms to assess secondary study outcomes including hospital admissions, ICU admissions, mechanical ventilator use, and mortality rates. During the study period, individuals with positive SARS-CoV-2 infections evaluated at our institution’s outpatient setting served as the reference population. Statistical analysis was performed using SPSS version24 (IBM Corp., Chicago, Illinois, USA, 2016); *P* < 0.05 was considered to indicate statistical signiﬁcance.

## RESULTS

A total of 58 individuals were enrolled in the study, with 27 allocated to the low-saline regimen and 28 to the high-saline regimen; three were lost to follow-up. There were no significant differences observed in either primary or secondary outcomes between the two arms (**Table 1**). Thus, gargling and nasal rinsing with either low- or high-saline solutions have equivalent effects in the durations of symptoms and equivalent clinical outcomes among individuals with SARS-CoV-2 infection. Compliance with the study protocol and the frequency of SARS-CoV-2 vaccination, Remdesivir, monoclonal antibodies, or Paxlovid use were also similar between the study groups.

**Table 1:** Demographic and clinical characteristics of the study population

Variables	Low-saline regimen (n = 27)*	High-saline regimen (n = 28)*	Reference population (n = 9398)*	*P*-value†	*P*-value‡	*P-*value§
Age (years)	39.0 (17–63)	41 (25–61)	45.0 (18–65)	0.43	0.32	0.76
BMI	29.6 (19–69)	31.7 (21–51)	30.0 (13–60)	0.35	0.28	0.56
Race				0.38	0.11	0.02
*Hispanic*	44.5 (12)	50.0 (14)	30.1 (2831)			
*Caucasian*	22.2 (6)	0.0	29.5 (2768)			
*African American*	18.5 (5)	28.6 (8)	3.9 (368)			
*Other*	14.8 (4)	14.3 (4)	36.5 (3431)			
Female	63.0 (17)	57.1 (16)	49.3 (4633)	0.66	0.16	0.41
Number of symptoms	7.0 (0–15)	5.0 (0–17)		0.97		
Largest duration of symptoms (days)	7.0 (0–14)	6.0 (0–14)		0.75		
Completed 14 d of treatment%	74.1 (20)	75.0 (21)		0.94		
SARS-CoV-2 vaccination rates¶	33.3 (9)	25.0 (7)	36.0 (3383)	0.46	0.77	0.23
Remdesivir treatment	7.4 (2)	17.9 (5)	22.2 (2086)	0.42	0.07	0.58
Monoclonal antibodies or Paxlovid treatment	3.7 (1)	7.1 (2)	1.5 (131)	0.57	0.31	0.01
Pneumonia	14.8 (4)	17.9 (5)	28.2 (2650)	0.76	0.12	0.23
Hospitalisation	18.5 (5)	21.4 (6)	58.8 (5530)	0.79	<0.001	<0.001
ICU admission	7.4 (2)	3.6 (1)	3.53 (332)	0.61	0.28	0.99
Mechanical ventilation	0.0	3.6 (1)	2.06(194)	0.32	0.55	0.57
Death	0.0	7.1 (2)	5.51 (518)	0.49	0.68	0.71

BMI – body mass index, SARS-CoV-2 – severe acute respiratory syndrome coronavirus-2, ICU – intensive care unit

*Data expressed as percentage (proportions) or median (range).

†Comparison between low and high-salt regimen.

‡Comparison between low-salt regimen and reference population;

§Comparison between high-salt regimen and reference population.

Symptoms: fever or chills, cough, sore throat, shortness of breath, difficulty breathing, headache, new loss of taste or smell, muscle or body aches, fatigue, nausea, vomiting, diarrhoea.

¶At least one dose of Moderna or Pfizer SARS-CoV-2 vaccines.

During the study period (August 2020–July 2022), our institution evaluated a total of 9398 individuals with positive SARS-CoV-2 infection (reference population, not including the study population). Notably, hospitalisation rates in the low-saline (18.5%) and high-saline (21.4%) regimens were significantly lower than in the reference population (58.8%; *P* < 0.001); however, no significant differences were noted in the rate of ICU admission.

## DISCUSSION

Our observations indicate that both low- and high- saline regimens for gargling and nasal rinsing are associated with similar frequencies and durations of symptoms related to SARS-CoV-2 infection. Notably, both saline regimens are linked to lower hospitalisation rates compared to individuals with SARS-CoV-2 who did not use these interventions.

A prior publication [7] conducted a secondary data analysis on a pilot randomised trial of hypertonic saline nasal irrigation and gargling for the common cold. In a post hoc analysis, the authors reported a reduction in the duration of alpha and beta coronavirus upper respiratory tract infections by an average of two and a half days, although their analysis did not include the SARS-CoV-2 virus. They acknowledged uncertainty regarding the effectiveness of hypertonic saline nasal irrigation and gargling against COVID-19 caused by SARS-CoV-2 [7]. Two recent randomised trials have shown that normal saline [3] or seawater [4] nasopharyngeal washes are associated with shorter symptom durations and reduced viral load in COVID-19 infections. In our study, we did not observe significant differences in symptom frequency or duration between individuals using either low- or high-saline regimens for gargling and nasal rinsing. However, we did note lower hospitalisation rates in both study arms compared to individuals infected with SARS-CoV-2 who did not use these interventions. However, these results should be taken with reservations given that the reference population was not predefined in the study design. Harris Health in Houston serves a large inner-city population with higher rates of chronic conditions including morbid obesity, diabetes, chronic hypertension and others. It is possible that these comorbidities might have contributed to the higher rates of hospitalisation among individuals with SARS-CoV-2 infection in the reference population.

The Centres for Disease Control and Prevention recommends gargling with salt water to soothe a sore throat caused by flu symptoms. The recommended solution is a mix of a cup of warm water with one teaspoon of salt. The recommendation is to gargle and then spit out the solution. A nested case-control study assessed the role of salt water gargling in treating various respiratory infections including clinically diagnosed tonsillitis, pharyngitis, laryngitis, sinusitis, otitis media, bronchitis, and pneumonia in addition to the use of antibiotics. The authors suggested that salt water gargling might help in the treatment of upper respiratory infections other than SARS-CoV-2 [8].

Although a pilot randomised trial of hypertonic saline nasal irrigation and gargling for the common cold showed promising results (such as lower use of over-the-counter medications, reduced transmission within the household and lower viral shedding), limitations in the study design were noted, such as lack of blinding and allocation concealment. The authors of the latter study speculated that the therapeutic effect of the hypertonic nasal irrigation and gargling was due to the chloride salts [5]. However, two randomised trials using plain water gargling also demonstrated beneficial effects [9,10].

Given the widespread availability of salt, our observations suggest that individuals with SARS-CoV-2 virus infection may benefit from low- or high- saline regimens for gargling and nasal rinsing, potentially leading to fewer hospital admissions. The risks associated with salt water gargling are minimal [11] and complications are rare [12]. Confirmation of these interventions’ effectiveness by larger studies is required to confirm that these interventions could alter the natural course of the COVID-19 disease process.

Proposed mechanism by which saline gargling and nasal rinsing may change the natural course of the COVID-19 disease includes limiting micro-aspiration of virus and secretions from the nasopharynx to deeper airways and lungs, direct effects on SARS-CoV-2 replication (impairment of growth and fusion in vitro), mucosal hydration, mucociliary clearance, altering the epithelial sodium channel activity, and preventing obstructive mucus/neutrophil extracellular trap formation [13].

Our study's small sample size may limit the generalisability and reliability of the results. Additional study limitations include our trial not being registered. The study duration probably encompassed multiple cycles of viral strains and therefore the two arms of the study may not be as directly comparable to the reference population. However, the risk for Hawthorne effect due to the possibility that the gargling group might have taken better care than the reference population should be lower because the researchers did not participate in the clinical care of these patients and the treating physicians were unaware of the inclusion of these patients into our study protocol. There is also the possibility of oversampling in the reference population given the duration of our study (two years). Our study’s strengths include its double-blind randomised control trial design and adequate power for the primary outcome. Additional studies addressing methodological shortcomings would be necessary to draw more definitive conclusions about the efficacy of saline regimens in managing symptoms of SARS-CoV-2 infection.

## CONCLUSIONS

Our findings suggest that low- and high- saline regimens for gargling and nasal rinsing are associated with similar symptom frequencies and durations in SARS-CoV-2 infection. Both regimens are also associated with lower hospitalisation rates compared to no saline nasopharyngeal wash in individuals infected by SARS-CoV-2. However, larger studies are needed to confirm these findings.
